# Clinic-based evaluation of the dual Xpert CT/NG assay on the GeneXpert System for screening for extragenital chlamydial and gonococcal infections amongst men who have sex with men

**DOI:** 10.1186/s12879-024-09042-4

**Published:** 2024-02-29

**Authors:** Maddalena Cordioli, Lorenzo Gios, Anna Erbogasto, Massimo Mirandola, Angela Sandri, Valeska Padovese, Carlos Caceres, Silver Vargas, Karel Blondeel, Ronaldo Silva, James Kiarie, Firdavs Kurbonov, Rosanna W. Peeling, Soe Soe Thwin, Daniel Golparian, Magnus Unemo, Igor Toskin, the ProSPeRo Network

**Affiliations:** 1 Infectious Diseases Section, Department of Medicine, Verona University Hospital, Verona, Italy; 2 WHO Collaborating Centre for Sexual Health and Vulnerable Populations - Epidemiology Unit - Division of Infectious Diseases, Verona University Hospital, Verona, Italy; 3 School of Sport and Health Sciences, University of Brighton, Brighton, UK; 4 Microbiology Section, Department of Diagnostics and Public Health, University of Verona, Verona, Italy; 5 Genitourinary Clinic, Department of Dermatology and Venereology, Mater Dei Hospital, Msida, Malta; 6 Centro de Investigación Interdisciplinaria en Sexualidad, Sida y Sociedad, Universidad Peruana Cayetano Heredia, Lima, Peru; 7 Department of Sexual and Reproductive Health and Research (includes the UNDP/UNFPA/UNICEF/WHO/World Bank Special Programme of Research, Development and Research Training in Human Reproduction [HRP]), World Health Organization, Geneva, Switzerland; 8 Faculty of Medicine and Health Sciences, Ghent University, Ghent, Belgium; 9 Department of Clinical Research, London School of Hygiene and Tropical Medicine, London, UK; 10 WHO Collaborating Centre for Gonorrhoea and Other STIs, Department of Laboratory Medicine, Faculty of Medicine and Health, Örebro University, Örebro, Sweden; 11 Institute for Global Health, University College London (UCL), London, UK

**Keywords:** *Neisseriagonorrhoeae*, *Chlamydiatrachomatis*, Gonococcal and chlamydial infections, Point-of-Care Tests (POCTs), Men who have Sex with Men, Public Health, diagnostic evaluation, Sexually transmitted infections

## Abstract

**Background:**

*Chlamydia trachomatis* (CT) and *Neisseria gonorrhoeae* (NG) infections have increased globally. Asymptomatic infections represent a significant risk of long-term complications. Men who have sex with men (MSM) are disproportionally affected, underscoring the need to offer screening programmes to this population. CT/NG Point of Care Testing (POCT) constitutes a strategic tool to improve the continuum of STI care, however extensive real-life evaluations amongst at risk populations are lacking. The aim of this study is to estimate the GeneXpert CT/NG assay performance and usability for CT and NG at genital and extragenital sites for screening amongst MSM.

**Methods:**

This study was a multi-site sexual health clinic-based evaluation (Italy, Malta and Peru) with consecutive enrolment. A first void urine sample (divided in two aliquots), two oropharyngeal and two anorectal swabs were collected for each study participant. One specimen set (one for each anatomical site) was tested with the dual index test (Cepheid) at the clinics by the healthcare staff, the other set with FDA/CE approved Nucleic Acid Amplification Tests (NAATs) at the laboratory. Clinical sites and reference laboratories participated in an internal and external quality control programme. Sensitivity, specificity, positive and negative likelihood ratios, positive and negative predictive values for each anatomical site were estimated using a meta-analytic approach.

**Results:**

One thousand seven hundred two MSM were recruited across all clinical sites for a total of 5049 biological specimens. NG and CT were respectively detected in 274 and 287 of samples. Overall, the NG POCT sensitivity and specificity was 91.43% and 99.75% in urine (LR + 372.80, LR- 0.09), 89.68% and 99.55% in rectal specimens (LR + 197.30, LR- 0.10) and 75.87% and 98.77% at the pharynx respectively (LR + 61.94, LR- 0.24). The CT component of the POCT sensitivity was 84.82% and specificity 99.63% in urine (LR + 228.68, LR- 0.15), 78.07% and 99.19% respectively on rectal site (LR + 96.23, LR-0.22), 67.79% and 99.88% respectively at pharyngeal site (LR + 554.89, LR- 0.32). 95.95% of MSM reported to be willing to wait for POCT results and no provider reported difficulties in terms of performance or interpretation of the results of the Xpert CT/NG.

**Conclusion:**

Rapid turnaround time, ease of use and high acceptability make the Xpert CT/NG testing system a strategic tool for increasing testing frequency, reaching those not yet tested and offering the possibility of immediate treatment if needed. The assay showed good negative likelihood ratios and confirms its use to rule out CT/NG infections. Sensitivity varied across sites and pathogens. Periodic staff training at the testing sites should be mandatory.

**Supplementary Information:**

The online version contains supplementary material available at 10.1186/s12879-024-09042-4.

## Background

According to the most recent WHO global estimates, four curable sexually transmitted infections (STIs) were responsible for 374 million cases in 2020 [1]. Among these, infection with *Chlamydia trachomatis* (CT) and *Neisseria gonorrhoeae* (NG) respectively accounted for an estimated 128 and 82 million cases [1]. Once detected, these two infections can be treated successfully with a short course of antibiotics. However, antibiotic resistance in NG has increased globally and concerns that it may become untreatable in certain circumstances have been raised [2, 3]. Many cases of both chlamydial and gonococcal infection are asymptomatic in both genital and extragenital sites (such as anorectal and oropharyngeal) representing a significant reservoir for infections spreading and enhancing the risk of HIV transmission if one of the partners has an untreated or not fully suppressed HIV infection [4–6]. In addition, if untreated, CT and NG can cause epididymitis, prostatitis and, although still under debate, infertility in the male population [7–9].

In the past decade, the prevalence rates of both CT and NG have been increasing amongst populations at risk, although they have varied significantly in terms of both type of infection and anatomical sites. In 2016 a systematic review assessed the prevalence of pharyngeal and anorectal CT and NG infection among women and men [10]. For men who have sex with men (MSM), the median prevalence of NG infection was 4.6% (min 0.5%, max 16.5%) in the pharynx and 5.9% (min 0.2%, max 24%) in the rectum, while for CT the median prevalence was 1.7% (min 0%, max 3.6%) and 8.9% (min 2.1%, max 23%) for the two anatomical sites respectively [10]. Other studies have shown that MSM carry a high burden of CT and NG [11, 12] and should be offered comprehensive targeted screening programmes [7, 13, 14], although, at the time the study commenced, there were no STI molecular diagnostics approved for detection of STI pathogens in extragenital anatomic sites. In addition, where they exist, screening programmes are far more common amongst women than men and are largely restricted to sampling genital sites [15]. To globally address this issue, the WHO in 2016 developed a strategy promoting the use of Point-Of-Care Tests (POCTs) and near-to-the patient tests as a tool for improving the continuum of STI services. The objective is to engage individuals as early as possible, retain them in care, minimizing loss to follow-up and guarantee appropriate treatment [16].

To better guide POCTs implementation, their performance characteristics and usability should be evaluated against gold standard laboratory-based tests. These evaluations should be conducted in a range of settings with different patient populations, disease prevalence and infrastructures [17]. Early POCTs for gonococcal and chlamydial infections such as lateral flow immunochromatographic tests (ICTs) or optical immunoassays (OIAs), have demonstrated poor performance, especially in terms of sensitivity [18–21]. They require a significant number of steps for sample preparation and test performance dependent on subjective human visual interpretation. More recently, CT/NG POCTs using nucleic acid amplification test (NAAT) technologies have been introduced [22–25]. Despite the high cost, these new diagnostic assays could expand their use in the field to include the detection of extra-genital NG and CT infections. A WHO laboratory validation study was carried out supporting the use of GeneXpert CT/NG POCT (hereafter termed as CT/NG POCT) for both urogenital, anorectal, and oropharyngeal specimens [26]. While the CT/NG POCT has been widely evaluated in clinical settings in genital anatomic sites in symptomatic men and women, less data are available on its performance when used to detect largely asymptomatic extragenital infections particularly when samples are collected and tested at peripheral clinical sites. The objective of this study was to determine the performance characteristics and usability of the GeneXpert® CT/NG assay on the GeneXpert® system (Cepheid) for screening for CT and NG at genital and extra-genital sites (anorectal and oropharyngeal) among MSM attending primary sexual health services when compared to gold standard laboratory based NAATs. In addition, the operational characteristics and acceptability of this near-patient technology to clients were assessed.

## Methods

### Study design

This clinic-based evaluation was a multi-site, observational study with a consecutive enrolment of MSM presenting at sexual health clinics for HIV/STI screening. Detailed study procedures and testing methodologies were based on a WHO standardised core protocol and have been presented elsewhere [27].

#### Study sites and population

The four study sites details (country, enrolment period, reference tests, participants, prevalence) are presented in Table 1. They were all STI-clinics for both general and key/vulnerable populations. The study protocol was approved by the Research Project Review Panel (RP2) of the WHO Department of Sexual and Reproductive Health and Research and by the WHO Ethics Review Committee (ERC) and locally by the site-specific ethics boards.

**Table 1:** Enrolment sites and reference testing in Italy, Malta, Peru

Country	Enrollment site	Start/End	Reference tests	Enrolled	Expected prevalence (%) for sample size calculation	
					**NG**	**CT**
Italy	Infectious Diseases and Tropical Medicine Unit	14-Feb-18; 14-May-19	COBAS 4800 NG/CT test	299	10	10
Malta	GU clinic, Mater Dei Hospital	20-Sep-19; 26-Feb-21	HOLOGIC Panther	742	9.2	11.1
Peru	Cerits Alberto Barton	16-Sep-19; 29-Jan-21	HOLOGIC DTS (in-house)	267	10	12
Peru	Centro Comunitario Epicentro	10-Sep-19; 20-Jan-21	HOLOGIC DTS (in-house)	394	10	12
**Total**				**1702**		

#### Inclusion / Exclusion criteria

The target population comprised MSM presenting at sexual health clinics asking for HIV/STI screening. The term MSM is used to describe those males who have sex with other males, regardless of whether or not they have sex with women or have a personal or social identity associated with that behaviour, such as being ‘gay’ or ‘bisexual’. MSM assigned male at birth of all gender identities were included in the study, information on gender identity was not collected.

All participants had to be at least 18 years old to participate.

MSM who refused to give consent, were younger than 18 years, and/or had previously participated in the study were excluded.

### Study procedure, specimen collection and testing procedures

Enrolled MSM received a unique study number. A structured questionnaire was used by all sites to collect data on demographics, behavioural characteristics, STI history and acceptability of the POCT approach. For each study participant, three types of specimens were collected: i) a sample of first void urine, ii) two oropharyngeal swabs collected by trained healthcare staff and iii) two anorectal swabs self-collected by enrolled respondents according to study standard operational procedures and manufacturer’s instructions. In addition, other biological samples were collected as part of routine clinical care. One swab per anatomical site and an aliquot of urine was used to perform the CT/NG POCT at the clinics, while the remaining swabs and urine specimen were sent to the reference laboratories. Swabs were collected in no particular order, and the manufacturer’s sample collection kits were used for the POCT and each commercial NAAT. At the laboratories, specimens that could not be processed immediately were stored at 4°C and processed within 4 days.

The GeneXpert Systems were installed at the clinics and samples were tested directly after collection by specifically trained clinic staff. Specimens generating an invalid, error or no POCT result were retested if adequate residual specimen was available. Results were recorded on a paper form.

Treatment was based on a site’s routine laboratory tests results. In the event of positive results of laboratory tests, participants received further information about the infection(s) and were treated following post-test counselling in line with international guidelines and local standards.

### POCT under evaluation

The test evaluated in this study was the GeneXpert CT/NG assay (Cepheid, Sunnyvale, California, United States) whose characteristics have been detailed elsewhere [27].

Although CT/NG POCT was originally approved for use with genital and urine specimens (first catch urine in men, urine, endocervical specimens and vaginal swab specimens in women), the U.S. Food and Drug Administration (FDA) cleared its use also with extragenital specimens (pharyngeal and anorectal swabs) in May 2019 [28].

### Reference laboratory tests

The laboratory NAATs used as reference for this study were both FDA and CE approved as required by the master protocol [29, 30].

In all reference laboratories, trained staff processed and tested the specimens according to the manufacturers’ instructions.

CT/NG assays from two manufacturers (performed on three different diagnostic systems) were used as laboratory-based reference NAATs (Table 1). Briefly, at the Italian reference laboratory, the cobas CT/NG assay on cobas 4800 system (Roche Diagnostics, Indianapolis, USA) was used. This assay uses real-time polymerase chain reaction (PCR) technology on a fully automated system for qualitative detection of CT and NG in patient specimens. Two different targets each for CT and NG are detected, i.e. to ensure high sensitivity and specificity [29–31].

The Aptima Combo 2 assay (Hologic/Gen-Probe Inc., San Diego, USA), detecting CT and NG, was performed on Tigris DTS system at the Peruvian central laboratory and on the Panther system at the WHO Collaborating Centre for Gonorrhoea and Other STIs in Sweden where the samples from Malta were tested. It has to be noticed that whereas the Panther system is fully automated, the DTS is semi-automated, requiring manual procedure from sample preparation to result reading. The Peruvian laboratory staff was specifically trained at the Hologic company in San Diego (CA) before the study started.

The Aptima Combo 2 assay is based on target capture of rRNA (23S rRNA for CT and 16S rRNA for NG) followed by transcription-mediated amplification (TMA) and a hybridization protection assay (HPA) for semi-quantitative detection of CT and NG [29, 31].

### Quality Assurance

Both the clinical sites and reference laboratories participated in internal quality control (IQC) and external quality assessment (EQA) programmes as detailed elsewhere [32].

IQC tests were conducted monthly using dry swabs with known bacterial loads (i.e. double negative, double positive) at the POCT sites. POCT and laboratory EQA tests were performed twice during the study period using a panel of 5 dry swab samples [32].

### Statistical Analysis

#### Sample size calculation

For NG/CT, a specificity of 98% and sensitivity of 80% was used to estimate the sample size for the study, with a 5% confidence interval around the point estimate. A conservative approach was adopted, assuming a POCT sensitivity lower than that both declared by the manufacturer [33] and proposed by WHO target product profiles for NG and CT POCTs [34]. The formula used for the sample size calculation, based on the 2006 WHO/TDR expert panel document on the evaluation of new diagnostic methods and techniques [35], yielded a target indicative recruitment for all sites of 245 positive results for both NG and CT.

#### Data analysis

Subjects’ demographic, STI-history and examination data, operational characteristics and acceptability of index tests data were summarised using descriptive statistics for aggregate and site level data. For all statistical tests the significance level was 5%. All statistical tests assumptions were assessed before carrying out the analysis and nonparametric alternative techniques were used in case of assumption violations. All analyses assumed that a true disease status by the reference test is known.

Sensitivity, specificity, positive and negative likelihood ratios (LR), positive predictive value (PPV) and negative predictive value (NPV) for the index test in each anatomical site were estimated by comparing the Xpert CT/NG results with those of the gold standard laboratory test [35, 36]. Test accuracies were reported following the Standards for Reporting Studies of Diagnostic Accuracy (STARD) guidelines [37].

A meta-analysis of diagnostic tests was used to analyse the results aggregating data from the four participating sites (Italy, Malta, Peru 1 and Peru 2). A Random Effects bivariate model with logit transformation was used to estimate pooled sensitivity, specificity, PPV, NPV, as well as likelihood ratio (LR) and diagnostic Odds Ratio accounting for between site heterogeneity. Hierarchical models were used to estimate tests diagnostic accuracy summary curve if thresholds for positivity vary across sites. Fixed effects models were used when there was low variability among sites because lack of convergence of the main model.

Predictive values were calculated from the summary sensitivity and specificity for a range of prevalence scenarios: the minimum and maximum of the observed sites’ prevalence and these values respectively minus (if possible) or plus 5%.

For data management, STATA Version 16.1 was used (College Station, TX: StataCorp LP) while SAS version 9.4 (SAS Institute Inc., Cary, NC, USA) was used for the statistical analysis.

## Results

### Demographic and Clinical Characteristics

A total sample of 1702 MSM were enrolled across all sites. The study participants had a pooled median age of 32 years (range 18–74), 56.8% of whom had a previous STI history. Syphilis was the most frequently reported previous STI (27.5%), followed by HIV (20.0%), gonorrhoea (19.4%) and chlamydial infection (14.8%); 18.8% reported other STIs, mostly viral infections such as anogenital herpes and/or HPV infection.

Almost a quarter (23.3%) of participants complained of STI symptoms at genital and/or extra-genital sites. Demographic characteristics of the enrolled population by geographical site is shown in Table 2. Participants’ clinical characteristics are presented as Supplementary material (Table 1S).

**Table 2:** Demographic characteristics of participants by site

Variable	Category	Overall		ITALY		MALTA		PERU (site 1)		PERU (site 2)	
Age	MIN	18		19		19		18		18	
	Q1	26		27		27		26		24	
	MEDIAN	32		34		33		32		28	
	Q3	40		46		40		42		34	
	MAX	74		74		69		69		61	
	Missing	0		0		0		0		0	
		N	%	N	%	N	%	N	%	N	%
Age	15–19	38	2.23	3	1.00	14	1.89	4	1.50	17	4.31
	20–24	264	15.51	33	11.04	83	11.19	50	18.73	98	24.87
	25–29	381	22.39	62	20.74	160	21.56	55	20.60	104	26.40
	30–34	341	20.04	58	19.40	158	21.29	46	17.23	79	20.05
	35–39	240	14.10	29	9.70	138	18.60	30	11.24	43	10.91
	40–44	164	9.64	31	10.37	79	10.65	27	10.11	27	6.85
	45–49	107	6.29	30	10.03	43	5.80	21	7.87	13	3.30
	50–54	78	4.58	19	6.35	31	4.18	19	7.12	9	2.28
	55–59	46	2.70	15	5.02	24	3.23	5	1.87	2	0.51
	60–64	26	1.53	9	3.01	7	0.94	8	3.00	2	0.51
	65–69	11	0.65	4	1.34	5	0.67	2	0.75	-	-
	70–74	6	0.35	6	2.01	-	-	-	-	-	-
Minutes to reach the site	MIN	2		5		NA		2		2	
	MEAN	39.64		37.77		NA		47.71		35.58	
	Q1	20		20		NA		20		20	
	MEDIAN	30		30		NA		30		30	
	Q3	45		45		NA		60		40	
	MAX	360		210		NA		360		180	

NA: info not available as not asked in the Maltese version of the questionnaire

missing values are not included in the denominator

### Results of the laboratory-based (reference) testing

A total of 5049 biological specimens from 1702 MSM were collected for laboratory evaluation. Laboratory test results for both NG and CT by enrolment site and anatomical site are detailed in Table 3. Overall, for NG 35 (2.1%) urine, 112 (6.7%) pharyngeal and 127 (7.5%) rectal samples were positive. For chlamydial infection, 54 (3.2%) urine, 45 (2.7%) pharyngeal and 188 (11.2%) rectal samples were positive.

**Table 3:** NG (A) and CT (B) positive and negative results according to laboratory reference tests by anatomical site

A												
Geographical site	N	Anatomical site	Missing	%	Invalid	%	Positive	%	Negative	%	Total	Prevalence (%)
Italy	299	Pharynx	1	0.3	0	-	12	4.0	286	95.7	299	4.0
		Rectum	0	-	1	0.3	15	5.0	283	94.6	299	5.0
		Urine	0	-	0	-	8	2.7	291	97.3	299	2.7
Malta	742	Pharynx	10	1.4	3	0.4	44	5.9	685	92.3	742	6.0
		Rectum	2	0.3	5	0.7	47	6.3	688	92.7	742	6.4
		Urine	30	4.0	1	0.1	16	2.2	695	93.7	742	2.3
Peru (site 1)	267	Pharynx	0	-	0	-	26	9.7	241	90.3	267	9.7
		Rectum	3	1.1	1	0.4	25	9.4	238	89.1	267	9.5
		Urine	1	0.4	0	-	6	2.2	260	97.4	267	2.3
Peru (site 2)	394	Pharynx	4	1.0	0	-	30	7.6	360	91.4	394	7.7
		Rectum	3	0.7	1	0.3	40	10.2	350	88.8	394	10.3
		Urine	3	0.7	1	0.3	5	1.3	385	97.7	394	1.3
Total	1702											
B												
Italy	299	Pharynx	1	0.3	0	-	5	1.7	293	98.0	299	1.7
		Rectum	0	-	2	0.7	19	6.4	278	93.0	299	6.4
		Urine	0	-	0	-	6	2.0	293	98.0	299	2.0
Malta	742	Pharynx	10	1.3	1	0.1	19	2.6	712	96.0	742	2.6
		Rectum	2	0.3	6	0.8	86	11.6	648	87.3	742	11.7
		Urine	30	4.0	4	0.6	26	3.5	682	91.9	742	3.7
Peru (site 1)	267	Pharynx	0	-	0	-	11	4.1	256	95.9	267	4.1
		Rectum	3	1.1	0	-	33	12.4	231	86.5	267	12.5
		Urine	1	0.4	0	-	16	6.0	250	93.6	267	6.0
Peru (site 2)	394	Pharynx	4	1.0	0	-	10	2.5	380	96.5	394	2.6
		Rectum	3	0.8	0	-	50	12.7	341	86.5	394	12.8
		Urine	3	0.8	0	-	6	1.5	385	97.7	394	1.5
Total	1702											

### POCT performance

Overall, 5035 samples were collected to be tested on the GeneXpert platform. Samples generating “Error” (10) or “Invalid” (14) for the dual CT/NG test, and for which no repeat test was done or for which the repeat test also resulted in error or invalid, were excluded from the analysis. Most of these excluded samples were from rectum, none were urine samples. Further details by geographical site and clinical sample are presented as Supplementary material (Table 2S and 3S).

### Detection of NG in specimens by POCT

In total, the POCTs yielded 36 (2.2%) NG-positive urine samples, 104 (6.2%) pharyngeal and 120 (7.1%) rectal samples. The highest percentage of NG-positives was from the rectal site in Peru at site 2 (10.2%), whereas the lowest for the same anatomical site was at the site in Italy (5.0%). As for pharynx the highest percentage of NG positive results was found in Peru at site 1 (9.7%) and the lowest in Italy (4.0%) while, for urine, the highest and the lowest was reported in Italy (2.7%) and Peru site 2 (1.3%) respectively.

**Fig. 1:**
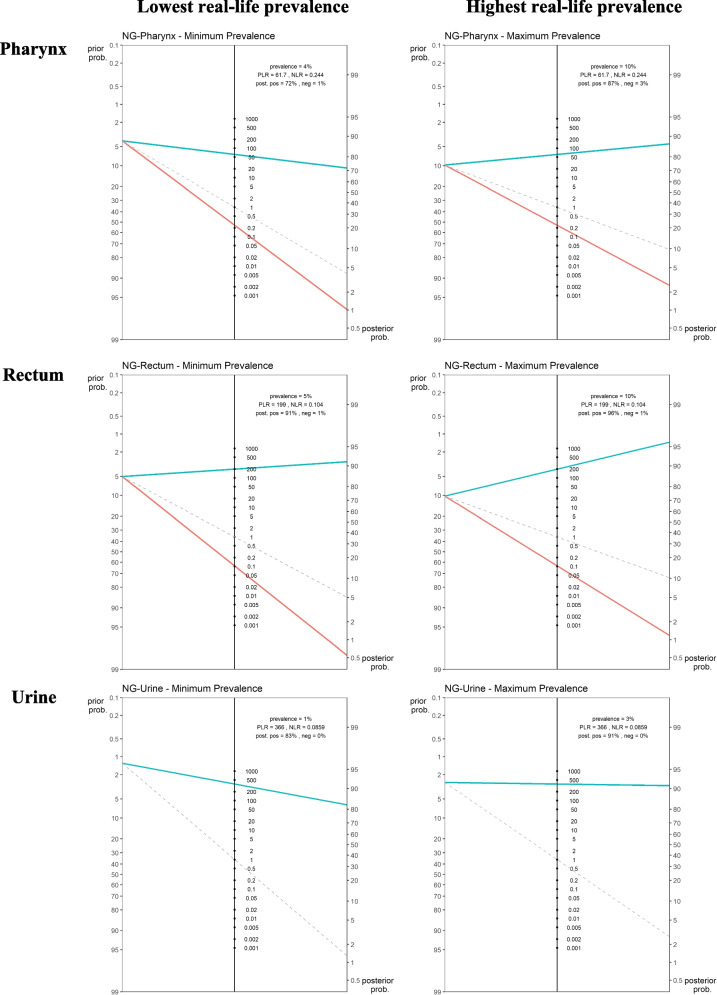
Fagan’s nomograms using real life lowest and highest *N. gonorrhoeae* prevalence per anatomical site (*y*-axis on the left represents the pre-test probability, *y*-axis on the right represents the post-test probability, aqua colour line indicates positive LR; red line indicates negative LR)

Overall, the POCT reached a sensitivity of NG detection in urine of 91.43% (CI 95% 76.54–97.21) and a specificity of 99.75% (CI 95% 99.35–99.91). The positive LR was 372.80 (CI 95% 139.19–998.51) while the negative LR was 0.09 (CI 95% 0.03–0.25). The sensitivity of POCT detection for NG in rectal specimens was 89.68% (CI 95% 83.03–93.92), with a specificity of 99.55% (CI 95% 99.05–99.78) (Table 4, Fig. 1S). The positive and negative LRs were 197.30 (CI 95% 93.91–414.52) and 0.10 (CI 95% 0.06–0.17) respectively. For the NG detection in pharyngeal specimens, the sensitivity was 75.87% (CI 95% 66.90–83.03) and the specificity 98.77% (CI 95% 97.94–99.27). The positive and negative LRs were 61.94 (CI 95% 36.42–105.33) and 0.24 (CI 95% 0.17–0.34) respectively (Table 4).

**Table 4:** Performance characteristics of POCTs for NG compared to reference assays (meta-analysis data)

		**Pharynx**		**Rectum**		**Urine**	
	**n**	**Sensitivity (%)**	**Specificity (%)**	**Sensitivity (%)**	**Specificity (%)**	**Sensitivity (%)**	**Specificity (%)**
Italy	**299**	75.00 (42.81–94.51)	98.25 (95.97–99.43)	100 (78.20–100)	99.64 (98.01–99.99)	87.5 (47.35–99.68)	100 (98.74–100)
Malta	**742**	84.09 (69.93–93.36)	99.42 (98.51–99.84)	85.11 (71.69–93.80)	99.42 (98.51–99.84)	93.75 (69.77–99.84)	99.71 (98.96–99.97)
Peru1	**267**	73.08 (52.21–88.43)	98.74 (96.38–99.74)	91.67 (73.00–98.97)	99.56 (97.58–99.99)	83.33 (35.88–99.58)	99.23 (97.25–99.91)
Peru2	**394**	66.67 (47.19–82.71)	98.05 (96.02–99.21)	90.00 (76.34–97.21)	99.71 (98.42–99.99)	100 (47.82–100)	100.00 (99.05–100.00)
**Fixed effects model**		75.89 (65.42–83.97)	98.79 (97.95–99.28)	89.68 (81.56–94.47)	99.55 (98.92–99.81)	91.43 (72.61–97.72)	99.75 (99.23–99.92)
**Random effects model**		75.87 (66.90–83.03)	98.77 (97.94–99.27)	89.68 (83.03–93.92)	99.55 (99.05–99.78)	91.43 (76.54–97.21)	99.75 (99.35–99.91)
		Between sites variability—SD (*p*-value)		Between sites variability—SD (*p*-value)		Between sites variability—SD (*p*-value)	
		7.08 (0.97)	7.19 (0.69)	-	-		
		DOR	253.57 (127.48–504.37)	DOR	1903.61 (743.87–4871.45)	DOR	4338.67 (930.91–20.221.17)
		LR +	61.94 (36.42–105.33)	LR +	197.30 (93.91–414.52)	LR +	372.80 (139.19–998.51)
		LR-	0.24 (0.17–0.34)	LR-	0.10 (0.06–0.17)	LR-	0.09 (0.03–0.25)

Considering the ranges of NG prevalence at the three anatomical sampling sites (1.28%—2.68% for urine, 4.3%—9.74% pharyngeal and 5.03%—10.26% rectal specimens), PPVs for different scenarios ranged from 74.19% to 96.82% in urine, from 65.84% to 91.43% in pharyngeal samples and from 86.16% to 97.29% in rectal samples (Table 5). The visual output of the POCT pre- and post- test probability, according to test’s positive and negative LRs as well as the gonococcal prevalence per anatomical site is shown in Fig. 1 using Fagan’s nomograms.

**Table 5:** NG prevalence scenarios using the minimum and maximum of the observed site prevalence and these values plus or minus 0.05 (when the subtraction of the fixed value from the minimum observed prevalence yielded a negative result, we reduce the fixed value to 0.01)

Pharynx					Rectum					Urine				
**Pooled Sensitivity**	**Pooled Specificity**	**Scenarios**	**PPV**	**NPV**	**Pooled Sensitivity**	**Pooled Specificity**	**Scenarios**	**PPV**	**NPV**	**Pooled Sensitivity**	**Pooled Specificity**	**Scenarios**	**PPV**	**NPV**
0,7587	0,9877	0,0303	0,6584	0,9924	0,8968	0,9955	0,0303	0,8616	0,9968	0,9143	0,9975	0,0078	0,7419	1,0000
		0,0403	0,7215	0,9898			0,0503	0,9135	0,9945			0,0128	0,8258	1,0000
**Prevalence range**		0,0974	0,8694	0,9743	**Prevalence range**		0,1026	0,9580	0,9883	**Prevalence range**		0,0268	0,9097	0,9999
0,0403	0,0974	0,1474	0,9143	0,9595	0,0503	0,1026	0,1526	0,9729	0,9817	0,0128	0,0268	0,0768	0,9682	0,9998

### Detection of CT in specimens by POCT

Overall, the POCTs yielded 52 (3.1%) CT-positive urine, 31 (1.8%) pharyngeal and 158 (9.4%) in rectal samples. The highest positivity rate in rectal specimens was recorded in Peru at site 2 (12.8%), the lowest in Italy (6.4%). As for pharyngeal samples, the highest CT positivity rate was found in Peru site 1 (4.1%) and the lowest in Italy (1.7%). For urine, the highest and the lowest rate was reported in Peru at site 1 (6.0%) and Peru at site 2 (1.5%) respectively.

**Fig. 2:**
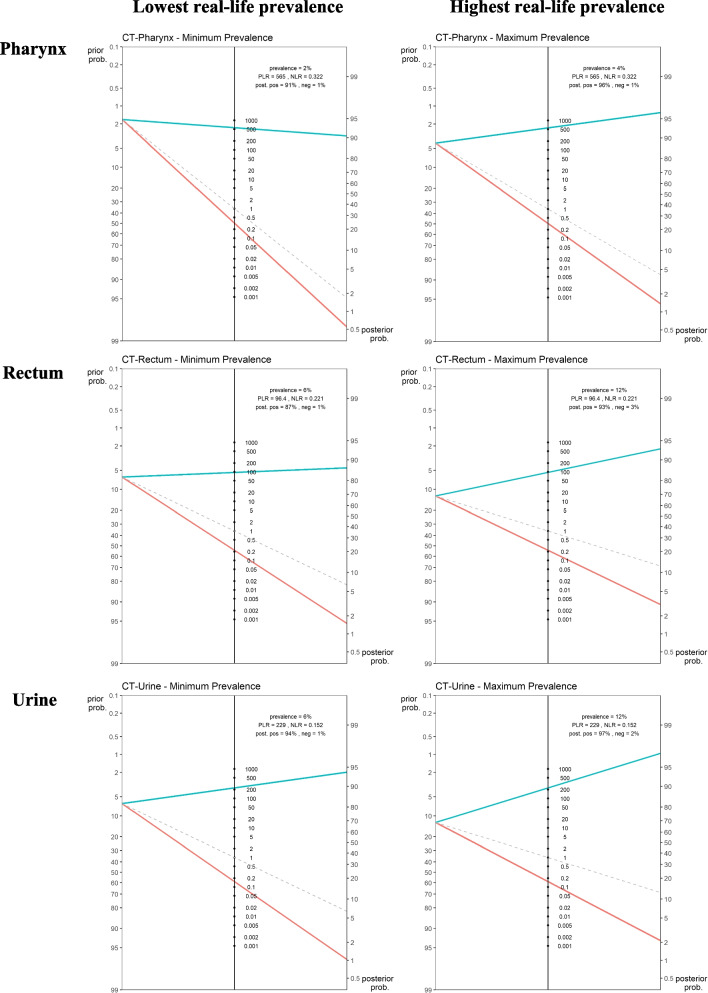
Fagan’s nomograms using real life lowest and highest *C. trachomatis* prevalence per anatomical site (*y*-axis on the left represents the pre-test probability, *y*-axis on the right represents the post-test probability, aqua colour line indicates positive LR; red line indicates negative LR)

For CT in urine, CT/NG POCT had a sensitivity of 84.82% (CI 95% 67.76–93.69) and a specificity of 99.63% (CI 95% 99.14–99.84). The positive and negative LRs were 228.68 (CI 95% 97.31–537.39) and 0.15 (CI 95% 0.07–0.35) respectively. On rectal swabs, sensitivity was 78.07% (CI 95% 71.57–83.44) and specificity 99.19% (CI 95% 98.58–99.54) (Table 6, Fig. 2S). The positive LR was 96.23 (CI 95% 54.46–170.03), the negative LR was 0.22 (CI 95% 0.17–0.29). At the pharyngeal site, the POCT had a sensitivity of 67.79% (CI 95% 42.92–85.49) and a specificity of 99.88% (CI 95% 99.51–99.97). The positive and negative LRs were 554.89 (CI 95% 133.34–2309.09) and 0.32 (CI 95% 0.16–0.65) respectively (Table 6).

**Table 6:** Performance characteristics of POCTs for CT compared to reference assays (meta-analysis data)

		**Pharynx**		**Rectum**		**Urine**	
	**n**	**Sensitivity (%)**	**Specificity (%)**	**Sensitivity (%)**	**Specificity (%)**	**Sensitivity (%)**	**Specificity (%)**
4001—Italy	**299**	100 (47.82–100)	100 (98.75–100)	100 (82.35–100)	98.53 (96.28–99.60)	83.33 (35.88–99.58)	98.98 (97.04–99.79)
4005—Malta	**742**	57.89 (33.50–79.75)	99.86 (99.22–100)	74.42 (63.87–83.22)	99.22 (98.20–99.75)	96.15 (80.36–99.90)	99.85 (99.19–100)
4006—Peru1	**267**	81.82 (48.22–97.72)	100 (98.56–100)	78.13 (60.03–90.72)	99.10 (96.77–99.89)	75.00 (47.62–92.73)	99.60 (97.79–99.99)
4007—Peru2	**394**	40.00 (12.16–73.76)	99.74 (98.54–99.99)	76.00 (61.83–86.94)	99.71 (98.38–99.99)	66.67 (22.28–95.67)	99.74 (98.56–99.99)
**Fixed effects model**		64.44 (46.92–78.80)	99.88 (99.38–99.98)	78.07 (70.32–84.26)	99.19 (98.43–99.58)	85.19 (70.39–93.29)	99.63 (99.05–99.85)
**Random effects model**		67.79 (42.92–85.49)	99.88 (99.51–99.97)	78.07 (71.57–83.44)	99.19 (98.58–99.54)	84.82 (67.76–93.69)	99.63 (99.14–99.84)
		Between sites variability—SD (*p*-value)		Between sites variability—SD (*p*-value)		Between sites variability—SD (*p*-value)	
		7.74 (0.64)	-	-	-	7.61 (0.60)	7.14 (0.94)
		DOR	1720.83 (305.54–9691.86)	DOR	435.33 (223.61–847.51)	DOR	1500.57 (412.30–5461.33)
		LR +	554.89 (133.34–2309.09)	LR +	96.23 (54.46–170.03)	LR +	228.68 (97.31–537.39)
		LR-	0.32 (0.16–0.65)	LR-	0.22 (0.17–0.29)	LR-	0.15 (0.07–0.35)

PPVs at genital site ranged from 78.08% (minimum observed prevalence 1.53%) to 93.62% (maximum observed prevalence 6.02%). At extragenital sites, PPVs ranged from 90.61% (min observed prevalence 1.68%) to 96.04% (max observed prevalence 4.12%) for pharynx and from 86.83% (min observed prevalence 6.4%) to 93.25% (max observed prevalence 12.53%) for rectum (Table 7). The visual output of the POCT's pre- and post- test probability, according to test’s positive and negative LRs as well as chlamydial prevalence per anatomical site, is shown in Fig. 2 using Fagan’s nomograms.

**Table 7:** CT prevalence scenarios using the minimum and maximum of the observed site prevalence and these values plus or minus 0.05 (when the subtraction of the fixed value from the minimum observed prevalence yielded a negative result, we reduce the fixed value to 0.01)

Pharynx					Rectum					Urine				
**Pooled Sensitivity**	**Pooled Specificity**	**Scenarios**	**PPV**	**NPV**	**Pooled Sensitivity**	**Pooled Specificity**	**Scenarios**	**PPV**	**NPV**	**Pooled Sensitivity**	**Pooled Specificity**	**Scenarios**	**PPV**	**NPV**
0,6779	0,9988	0,0068	0,7946	0,9978	0,7807	0,9919	0,0140	0,5778	0,9969	0,8482	0,9963	0,0103	0,7046	1,0000
		0,0168	0,9061	0,9945			0,0640	0,8683	0,9851			0,0153	0,7808	0,9999
**Prevalence range**		0,0412	0,9604	0,9863	**Prevalence range**		0,1253	0,9325	0,9693	**Prevalence range**		0,0602	0,9362	0,9997
0,0168	0,0412	0,0912	0,9827	0,9687	0,0640	0,1253	0,1753	0,9535	0,9551	0,0153	0,0602	0,1102	0,9660	0,9995

### Acceptability

Across all testing sites, a large majority of study participants were, in principle, willing to have specimens tested using a POCT and wait at the testing site to receive their results (95.95% *n* = 1633). As for the waiting time, 22.41% were willing to wait up to two hours, 41.58% up to one hour and 30.8% up to 30 min (Table 3S as Supplementary material).

### Operational characteristics

Over three quarters of providers found the instructions for use very clear (43%) or excellent (36%), none thought they were difficult. Providers did not report any difficulty in the interpretation of the results. Over three quarters also indicated hands on time was 10 min (43%) or less (36%) and no provider indicated the use as complicated. 79% of providers indicated 30 to 60 min as optimal training time.

## Discussion

CT and NG infections are becoming increasingly prevalent amongst MSM. Considering that the majority of these infections are asymptomatic, screening should be carried out at extragenital sites with reliable and accurate screening tests for extragenital specimens [7]. In fact, over the last two decades, the screening for NG and CT infections has become easier mainly due to the introduction of NAATs that currently represent the diagnostic golden standard [29, 30].

Unfortunately, laboratory-based NAATs can be very complex, expensive and are not widely available in many healthcare settings, particularly in low- and middle-income countries (LMICs) [38]. As a consequence, these laboratory tests are not widely accessible to the majority of people in the world [38] and, therefore, only a limited number of individuals can benefit from this high standard of diagnosis and subsequent treatment. This limits the possibility of an equitable coverage of services, particularly for disadvantaged and stigmatized populations. To overcome this disparity, in 2016 the WHO reaffirmed the importance of the POCT approach in improving the continuum of STI services provision with the aim of engaging individuals as early as possible, retaining them in care and minimizing any loss to follow-up [16]. The development of the GeneXpert platform and other potential near-patient platforms has enhanced the possibility of providing NAAT diagnostics at POC, thus permitting appropriate treatment at an initial consultation.

The study reported here is part of a wider international evaluation of POCTs and near-patient technologies for the diagnosis of the most prevalent and clinically challenging STIs conducted by WHO and its international partners (The ProSPeRo Network) [39]. In this study, we evaluated the analytical performance characteristics of the Xpert CT/NG assay compared to laboratory-based NAATs and the acceptability of the near-patient platform to end users when used to screen for NG and CT in both genital and extra-genital anatomical sites among MSM attending sexual health clinics in Italy, Malta and Peru. To the best of our knowledge, all previous evaluation studies, even if they were based on prospectively collected clinical specimens, were aimed at evaluating the performance of GeneXpert system in specialized laboratories. In our study, the instruments were made available at the clinics where patients were evaluated and the testing was performed on specimens directly after their collection by non-laboratory clinic staff.

The results reported here show reasonably good agreement with those previously reported in laboratory-based evaluation studies on genital specimens [20, 21, 40]. Considering male urine, in our real-life clinical study, the Xpert sensitivity was 91.43% (CI 95% 76.54–97.21) for NG, that resulted slightly lower compared to the laboratory evaluation (98%—CI 95% 88.4–99.9) [21]. For CT, the sensitivity was found significantly lower (84.82%—CI 95% 67.76–93.69) compared to figures previously published (97.5%—CI95% 91.4% to 99.7%) [20]. Despite these differences in sensitivity, the PPVs resulted above 90% with the highest observed prevalence for NG (2.68%, PPV 90.97%) and CT (6.02%, PPV 93.62%).

As for CT/NG POCT performance on pharyngeal swab, our findings are in line with a meta-analysis published by Bristow and Colleagues in 2019 [41]. Estimated with a Bayesian approach, in their paper the positive per cent agreement ranged from 50 to 100% for CT and from 77.8% to 97.3% for NG [41]. In our study, the sensitivity was 67.79% (CI95% 42.92–85.49), and 75.87% (CI95% 66.90–83.03) for chlamydial and gonococcal infection respectively. Considering prevalence scenarios, even with a minimum observed prevalence of 1.68% the PPV for CT was above 90%, whereas for NG this value was reached only with the highest prevalence scenario (14.74%, PPV 91.43%). This may result in a significant number of undiagnosed infections if the diagnosis is based solely on test results from a single extragenital site.

As for the anorectal site, where both gonococcal and chlamydial infections were more frequently detected, the sensitivity point estimates of our study are lower than those already published [42, 43] although looking at the confidence interval they fall within the 95%CI of all studies. In particular, our estimates were 78.07% (95%CI 71.57–83.44) and 89.68% (83.03–93.92) for CT and NG respectively, substantially in line with those described by Goldenberg et al. (CT: 86%—95%CI 72.1–94.7; NG: 91.1%—95% CI, 80.4–97) [42].

When positive predictive values are considered, while NG was consistently above 90% even in the lowest observed prevalence (5.03%, PPV 91.35%), CT detection showed a PPV of 93.25% only when the highest observed prevalence (12.53%) was applied.

In line with the literature, the CT/NG Xpert specificity for both pathogens in all anatomical sites was very high with the lowest estimate of 98.77% (95%CI 97.94–99.27) for NG at the pharynx. Also considering the prevalence scenarios, the NPVs were consistently above 95%, with the lowest value for CT at rectal site when the highest prevalence (95.51% with 17.53% prevalence) was used.

There may be several reasons to explain the suboptimal performance of the Xpert CT/NG assay reported in our evaluation when compared with laboratory reference NAATs.

The real-life setting, with the majority of the samples collected from asymptomatic individuals, might have had an impact on index test performance as many published results are based on evaluations conducted using clinical specimens that are from symptomatic individuals [40, 42–44], which may have a higher bacterial load than asymptomatic patients. In addition, the GeneXpert platform was used in a laboratory setting by well-trained laboratory staff that might have lowered the chance of human error and therefore have increased the POCT performance [40, 42–44]. Our study is one of the few, if not the only one, in which the GeneXpert system was evaluated in a non-laboratory setting by non-laboratory technicians (although specifically trained by representatives of the manufacturer) and this might have had an impact on test performance evaluation. However, the study methodology was specifically designed to guarantee the correct implementation of testing procedures in a non-lab setting. The EQA results across all study sites confirm that procedures were followed consistently [32]. In addition, the healthcare staff involved in the study was properly trained by Cepheid and WHO trainers in the proper use of the GeneXpert system. Clinic staff followed manufacturer’s instructions for both specimen collection and testing. In addition, internal and external quality controls were routinely performed during the whole study period, ensuring that no systematic errors in the Xpert CT/NG procedures were made. Notwithstanding these discrepancies in sensitivity, it is important to describe POCT performance in terms of likelihood ratio and test efficacy rather than in terms of sensitivity and specificity alone [45].

Another factor that may have impacted POCT sensitivity might be due to anorectal self-sampling, but the literature suggests the same quality as healthcare provider collection) [46].

An additional factor that may have impacted on the sensitivity could be the moderate sample size of positive specimens that is also reflected in the confidence intervals although, for the sample size of the study, a conservative approach was used considering 80% sensitivity of the CT/NG Xpert, which is lower than the one declared by the manufacturer [33] and published in other studies [40, 41].

The study confirms that a CT/NG POCT negative result can exclude the infections and therefore guide clinicians as to whether to prescribe additional tests and/or antibiotics. The high negative likelihood ratio of the Xpert CT/NG assay can rule out the presence of both infections regardless of the anatomical site (Figs. 1 and 2).

As for the positive likelihood ratio and the positive post-test probability, these vary largely across anatomical sites and infection prevalence. Considering that, as stated by WHO in its POCT target product profile [34], the minimal acceptable sensitivity for POCT is 90% for NG and higher than 90% for CT, our study shows that the Xpert CT/NG assay seems to have adequate performance for NG only in rectal specimens (Fig. 1) while for CT, this is the case in urine and pharyngeal swabs (Fig. 2). However, when highest infection prevalence scenarios are considered, for both NG and CT, a positive POCT result proved to be more reliable for all anatomical sites, with the only exception of NG at the pharynx where the positive post-test probability resulted slightly lower than 90% (Figs. 1 and 2).

Diagnostic tools should be considered and evaluated not only in terms of analytical characteristics, but in the broader context of clinical utility [47]. Based on anatomical sites and country prevalence scenarios, clinicians and public health officers can better understand the meaning and the limitations of the POCT in guiding resource allocations, diagnostic pathways and antibiotic prescriptions.

### NG and CT prevalence

In the past ten years, many international guidelines have recommended both genital and extragenital screening for NG and CT amongst asymptomatic MSM [7, 13, 14, 48, 49] and, in this context, the WHO endorsed the use of POCTs to improve screening strategies and reach those never or not sufficiently tested for STIs [16].

Testing strategies are based on infection and AMR prevalence, proportion of symptomatic infections, long-term consequences at the individual level and transmission rate. Both CT and NG infections are often asymptomatic and highly transmittable and many studies have demonstrated that a significant proportion of infections would be missed if only genital sites were tested [10, 50, 51], particularly for specific populations such as MSM.

In the most recent literature, however, there is a lack of consensus on NG/CT testing frequency and its consequences [52, 53]. In fact, according to Kenyon, extensive screening and subsequent treatment may be linked to a temporary decrease in bacterial STI prevalence, but an increase of resistant strains due to a much higher use of antibiotics [53].

Across the geographical sites, the prevalence rates of CT and NG infection found in our study appear to be consistent with those among MSM reported elsewhere [10, 51]. It should be noted that previous studies that reported higher NG and CT rates of infection enrolled many symptomatic subjects [54–56]. If MSM are sampled for NG and CT from the urethral site only (using first void urine or urethral swabs), a significant number of rectal and pharyngeal CT/NG infections would be missed [10, 50, 51] which increases the transmission risk of HIV if one partner has an undiagnosed or not fully suppressed infection and therefore perpetuating a reservoir of CT/NG that potentially further spreads these infections in the community. It is also important to bear in mind that, considering the infection and coinfection rates of CT and NG amongst MSM [51], the availability of a valid dual CT/NG POCT allows the concomitant screening for both infections with just a single cartridge and a subsequent reduced impact of screening costs.

## Conclusions

Rapid turnaround time and ease of use make the Xpert® CT/NG testing system well suited for near-patient testing offering the possibility of immediate treatment if needed.

The assay showed optimal specificity regardless of the type of infection and anatomical site. In particular, our findings showed very good negative LRs and confirm the use of Xpert CT/NG to rule out further assessment in the case of a negative result regardless of the prevalence of infection. Sensitivity varied across sites and pathogens, with the highest agreement with the reference test reached on urine for NG and lowest on pharynx for CT. Given the high acceptability of the Xpert CT/NG assay by our target population, it could be a strategic tool for implementing testing frequency and reaching those not yet tested in both traditional and unconventional testing venues. However, as in our real-life clinical study, the use of STI POCTs in non-laboratory settings and by non-laboratory staff has to be conceived in the context of a cyclic training and quality monitoring programme with the aim of guaranteeing the optimal quality standard required for all diagnostic tests.

Finally, considering the findings of our study, further efforts should be made to reduce the accessing time to CT/NG POCT results.

### Supplementary Information

**Additional file:** **Table 1S.** Clinical characteristics of participants by site. **Table 2S.** NG positive and negative results according to POCT. **Table 3S.** CT positive and negative results according to POCT. **Table 4S.** Time study participants are willing to wait for POCT result, by geographical site.** Figure 1S.** Performance characteristics of Xpert assay for *Neisseria gonorrhoeae* compared to reference assays (by anatomical and geographical site). **Figure 2S.** Performance characteristics of Xpert assay for *Chlamydia trachomatis* compared to reference assays (per site).

## Data Availability

This study which forms part of a larger programme of research co-ordinated by WHO and for which WHO acts as the repository of the ensemble of the results obtained from the individual projects. In view of this, all rights to the results of the study hold and exercise patent rights in respect of any invention resulting from the study, are the subject of co-ownership and responsibility between the WHO and respective country sites. Dr Igor Toskin is the Chief Investigator and contact for data availability queries (toskini@who.int).
