# Supplementary material for: Clinic-based evaluation of the dual Xpert CT/NG assay on the GeneXpert System for screening for extragenital chlamydial and gonococcal infections amongst men who have sex with men
Source: BMC Infect Dis. 2024 Feb 29;24(Suppl 1):224. doi: 10.1186/s12879-024-09042-4 (PMC10902931; doi:10.1186/s12879-024-09042-4)
Supplement: Supplementary file 1 — Additional file: Table 1S. Clinical characteristics of participants by site. Table 2S. NG positive and negative results according to POCT. Table 3S. CT positive and negative results according to POCT. Table 4S. Time study participants are willing to wait for POCT result, by geographical site. Figure 1S. Performance characteristics of Xpert assay for Neisseria gonorrhoeae compared to reference assays (by anatomical and geographical site). Figure 2S. Performance characteristics of Xpert assay for Chlamydia trachomatis compared to reference assays (per site). [file 12879_2024_9042_MOESM1_ESM.docx]

**TABLES AND FIGURES (SUPPLEMENTARY MATERIAL)**

Table 1S: Clinical characteristics of participants by site

| **Variable** | **Category** | **Overall** | | **ITALY** | | **MALTA** | | **PERU (site 1)** | | **PERU (site 2)** | |
| --- | --- | --- | --- | --- | --- | --- | --- | --- | --- | --- | --- |
| **Previous STIs diagnosis (self-reported)** | | N | % | N | % | N | % | N | % | N | % |
|  | No | 726 | 43.24 | 123 | 41.14 | 329 | 45.44 | 126 | 47.55 | 148 | 37.85 |
|  | Yes | 953 | 56.76 | 176 | 58.86 | 395 | 54.56 | 139 | 52.45 | 243 | 62.15 |
|  | Missing | 23 |  | 0 |  | 18 |  | 2 |  | 3 |  |
| Gonorrhoea | No | 1331 | 79.58 | 239 | 79.93 | 530 | 73.5 | 228 | 86.04 | 334 | 85.42 |
|  | Yes | 335 | 19.72 | 59 | 19.73 | 187 | 25.94 | 35 | 13.20 | 54 | 13.81 |
|  | Don't know/Don't want to answer | 10 | 0.71 | 1 | 0.33 | 4 | 0.55 | 2 | 0.75 | 3 | 0.77 |
|  | Missing | 26 |  | 0 |  | 21 |  | 2 |  | 3 |  |
| Chlamydia | No | 1442 | 85.94 | 264 | 88.29 | 549 | 75.93 | 253 | 95.47 | 356 | 91.05 |
|  | Yes | 248 | 14.78 | 34 | 11.37 | 172 | 23.79 | 11 | 4.15 | 31 | 7.92 |
|  | Don't know/Don't want to answer | 8 | 0.48 | 1 | 0.33 | 2 | 0.28 | 1 | 0.38 | 4 | 1.02 |
|  | Missing | 24 |  | 0 |  | 19 |  | 2 |  | 3 |  |
| HIV | No | 1338 | 79.88 | 234 | 78.26 | 631 | 87.64 | 199 | 75.09 | 274 | 70.08 |
|  | Yes | 335 | 20.00 | 65 | 21.74 | 89 | 12.36 | 66 | 24.91 | 115 | 29.41 |
|  | Don't know/Don't want to answer | 2 | 0.12 | 0 | - | 0 | - | 0 | - | 2 | 0.51 |
|  | Missing | 27 |  | 0 |  | 22 |  | 2 |  | 3 |  |
| Syphilis | No | 1213 | 72.72 | 210 | 70.23 | 595 | 82.64 | 161 | 60.75 | 247 | 63.17 |
|  | Yes | 460 | 27.46 | 89 | 29.77 | 125 | 17.36 | 104 | 39.25 | 142 | 36.32 |
|  | Don't know/Don't want to answer | 2 | 0.12 | 0 | - | 0 | - | - | - | 2 | 0.51 |
|  | Missing | 27 |  | 0 |  | 22 |  | 2 |  | 3 |  |
| Other STIs | No | 1344 | 81.01 | 226 | 75.59 | 551 | 76.53 | 261 | 98.49 | 306 | 78.66 |
|  | Yes | 311 | 18.75 | 73 | 24.41 | 154 | 21.39 | 4 | 1.50 | 80 | 20.57 |
|  | Don't know/Don't want to answer | 4 | 0.24 | 0 | - | 1 | 0.14 | - | - | 3 | 0.77 |
|  | Missing | 43 |  | 0 |  | 36 |  | 2 |  | 5 |  |
|  |  |  |  |  |  |  |  |  |  |  |  |
| **Antibiotic treatment in the 3 weeks before enrolment** | | | |  |  |  |  |  |  |  |  |
|  | No | 1382 | 81.29 | 254 | 84.95 | 657 | 88.78 | 180 | 67.42 | 291 | 73.86 |
|  | Yes | 309 | 18.18 | 44 | 14.72 | 82 | 11.08 | 86 | 32.21 | 97 | 24.62 |
|  | Not aware | 5 | 0.29 | - | - | - | - | 1 | 0.37 | 4 | 1.02 |
|  | Don't know | 3 | 0.18 | 1 | 0.33 | 1 | 0.14 | - | - | 1 | 0.25 |
|  | Don't want to answer | 1 | 0.06 | - | - | - | - | - | - | 1 | 0.25 |
|  | Missing | 2 |  | 0 |  | - | - | 0 |  | 0 |  |
|  |  |  |  |  |  |  |  |  |  |  |  |
| **STIs symptoms** | | | | | | | | | | | |
|  | No | 1280 | 76.60 | 241 | 80.87 | 531 | 74.58 | 187 | 70.04 | 321 | 81.47 |
|  | Yes | 391 | 23.40 | 57 | 19.13 | 181 | 25.42 | 80 | 29.96 | 73 | 18.53 |
|  | Missing | 31 |  | 1 |  | 30 |  | 0 |  | 0 |  |
|  |  |  |  |  |  |  |  |  |  |  |  |
| If yes, in which anatomical site(s)? | Urethral | 119 | 30.43 | 22 | 38.60 | 51 | 28.18 | 40 | 50.00 | 6 | 8.22 |
|  | Rectal | 98 | 25.06 | 14 | 24.56 | 40 | 22.10 | 28 | 35.00 | 16 | 21.92 |
|  | Urethral and rectal | 33 | 8.44 | 8 | 14.03 | 10 | 5.52 | 12 | 15.00 | 3 | 4.11 |
|  | Other site and/or systemic symptoms | 141 | 36.06 | 13 | 22.81 | 80 | 44.20 | 0 |  | 48 | 65.75 |

*Note: missing values are not included in the denominator*

Table 2S: NG positive and negative results according to POCT.

| **Geographical site** | **Number of enrolled subjects** | **Anatomical site** | **Error** | **%** | **Invalid** | **%** | **Positive** | **%** | **Negative** | **%** | **Total** |
| --- | --- | --- | --- | --- | --- | --- | --- | --- | --- | --- | --- |
| Italy | 299 | Pharynx | 0 | - | 0 | - | 14 | 4.7 | 284 | 95.3 | 298 |
|  |  | Rectum | 6 | 2.0 | 0 | - | 16 | 5.4 | 276 | 92.6 | 298 |
|  |  | Urine | 0 | - | 0 | - | 7 | 2.3 | 292 | 97.7 | 299 |
| Malta | 742 | Pharynx | 0 | - | 1 | 0.1 | 41 | 5.6 | 687 | 94.3 | 729 |
|  |  | Rectum | 2 | 0.3 | 1 | 0.1 | 44 | 6.0 | 688 | 93.6 | 735 |
|  |  | Urine | 0 | - | 0 | - | 17 | 2.4 | 694 | 97.6 | 711 |
| Peru (site 1) | 267 | Pharynx | 1 | 0.4 | 1 | 0.4 | 22 | 8.2 | 243 | 91.0 | 267 |
|  |  | Rectum | 1 | 0.4 | 10 | 3.8 | 23 | 8.7 | 229 | 87.1 | 263 |
|  |  | Urine | 0 | - | 0 | - | 7 | 2.6 | 259 | 97.4 | 266 |
| Peru (site 2) | 394 | Pharynx | 0 | - | 1 | 0.3 | 27 | 6.9 | 362 | 92.8 | 390 |
|  |  | Rectum | 0 | - | 0 | - | 37 | 9.5 | 353 | 90.5 | 390 |
|  |  | Urine | 0 | - | 0 | - | 5 | 1.3 | 385 | 98.7 | 390 |
| Overall | 1702 | Pharynx | 1 | 0.1 | 3 | 0.2 | 104 | 6.2 | 1576 | 93.6 | 1684 |
|  |  | Rectum | 9 | 0.5 | 11 | 0.7 | 120 | 7.1 | 1546 | 91.7 | 1686 |
|  |  | Urine | 0 | - | 0 | - | 36 | 2.2 | 1630 | 97.8 | 1666 |

Figure 1S: Performance characteristics of Xpert assay for *Neisseria gonorrhoeae* compared to reference assays (by anatomical and geographical site).

| **Pharynx** | 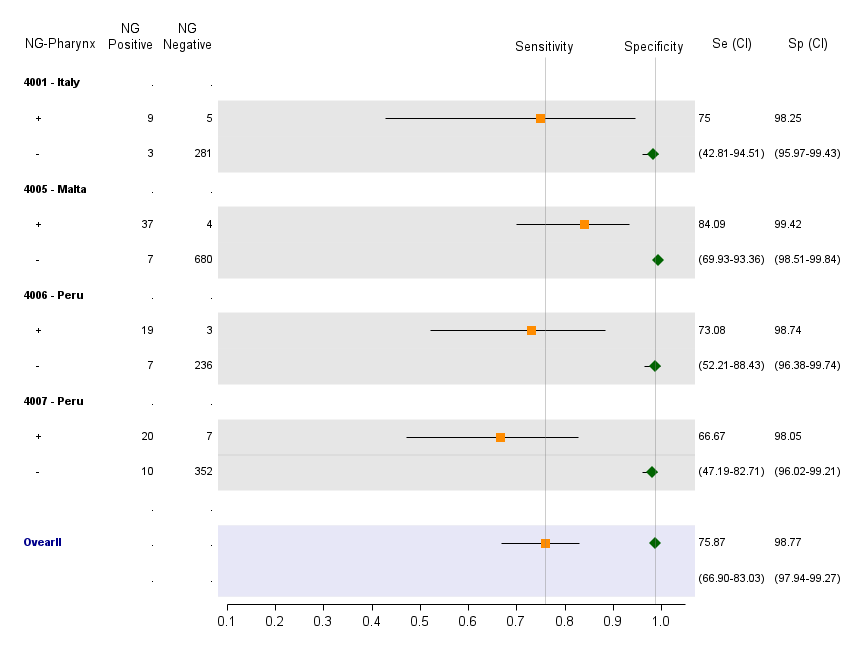 |
| --- | --- |
| **Rectum** | 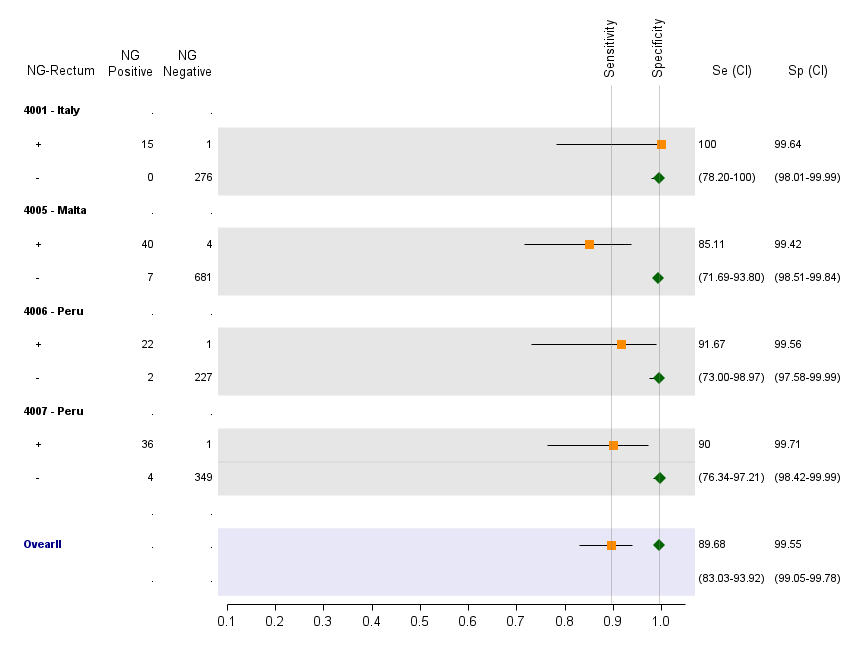 |
| **Urine** | 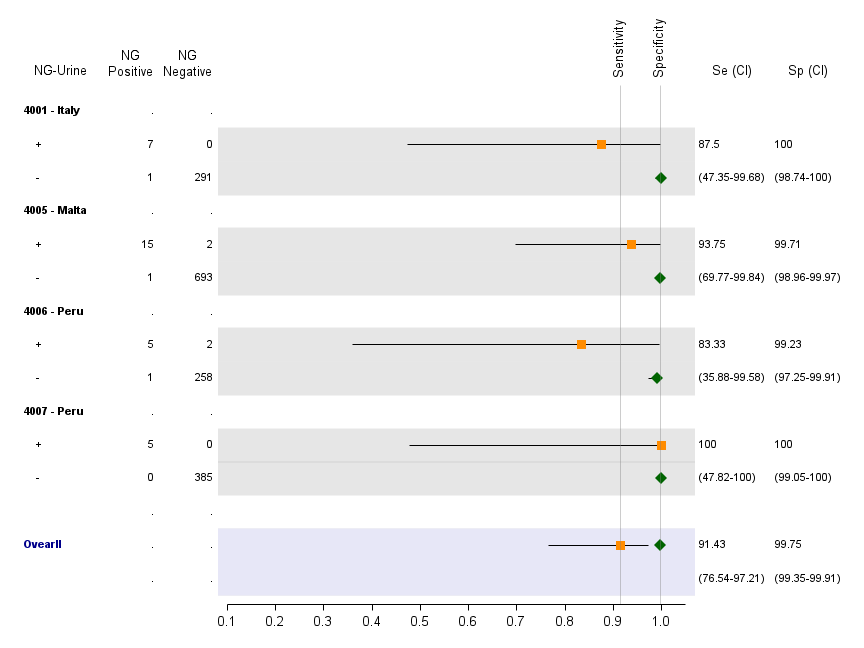 |

Table 3S: CT positive and negative results according to POCT.

| **Geographical site** | **Number of enrolled subjects** | **Anatomical site** | **Error** | **%** | **Invalid** | **%** | **Positive** | **%** | **Negative** | **%** | **Total** |
| --- | --- | --- | --- | --- | --- | --- | --- | --- | --- | --- | --- |
| Italy | 299 | Pharynx | 0 | - | 0 | - | 5 | 1.7 | 293 | 98.3 | 298 |
|  |  | Rectum | 6 | 2.0 | 0 | - | 23 | 7.7 | 268 | 90.3 | 297 |
|  |  | Urine | 0 | - | 0 | - | 8 | 2.7 | 291 | 97.3 | 299 |
| Malta | 742 | Pharynx | 0 | - | 1 | 0.1 | 12 | 1.6 | 718 | 98.2 | 731 |
|  |  | Rectum | 2 | 0.3 | 1 | 0.1 | 69 | 9.4 | 662 | 90.2 | 734 |
|  |  | Urine | 0 | - | 0 | - | 26 | 3.7 | 682 | 96.3 | 708 |
| Peru (site 1) | 267 | Pharynx | 1 | 0.4 | 1 | 0.4 | 9 | 3.4 | 256 | 95.9 | 267 |
|  |  | Rectum | 1 | 0.4 | 10 | 3.8 | 27 | 10.2 | 226 | 85.6 | 264 |
|  |  | Urine | 0 | - | 0 | - | 13 | 4.9 | 253 | 95.1 | 266 |
| Peru (site 2) | 394 | Pharynx | 0 | - | 1 | 0.3 | 5 | 1.3 | 384 | 98.5 | 390 |
|  |  | Rectum | 0 | - | 0 | - | 39 | 10.0 | 352 | 90.0 | 391 |
|  |  | Urine | 0 | - | 0 | - | 5 | 1.3 | 386 | 98.7 | 391 |
| Overall |  | Pharynx | 1 | 0.1 | 3 | 0.2 | 31 | 1.8 | 1651 | 97.9 | 1686 |
|  |  | Rectum | 9 | 0.5 | 11 | 0.7 | 158 | 9.4 | 1508 | 89.4 | 1686 |
|  |  | Urine | 0 | - | 0 | - | 52 | 3.1 | 1612 | 96.9 | 1664 |

Figure 2S: Performance characteristics of Xpert assay for *Chlamydia trachomatis* compared to reference assays (per site).

| **Pharynx** | 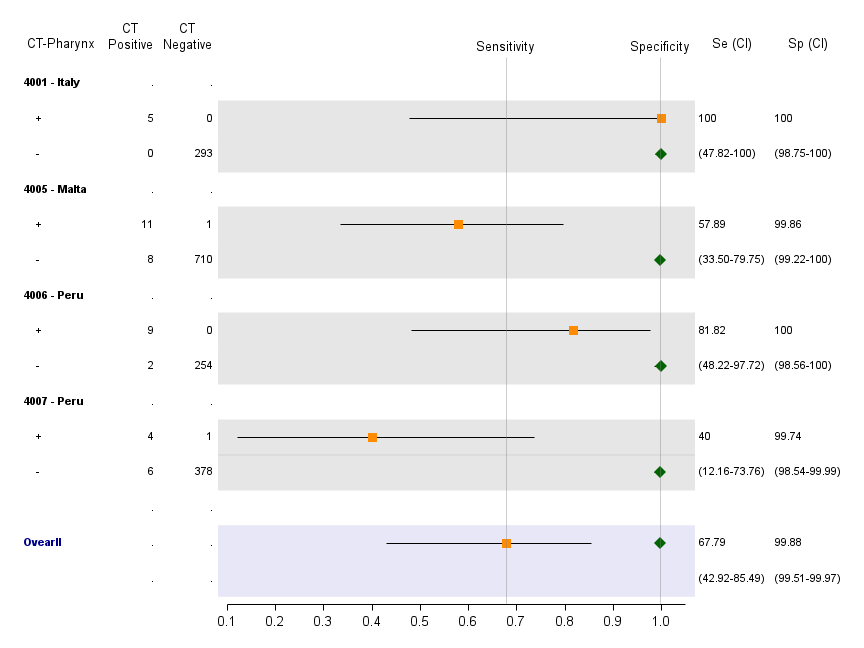 |
| --- | --- |
| **Rectum** | 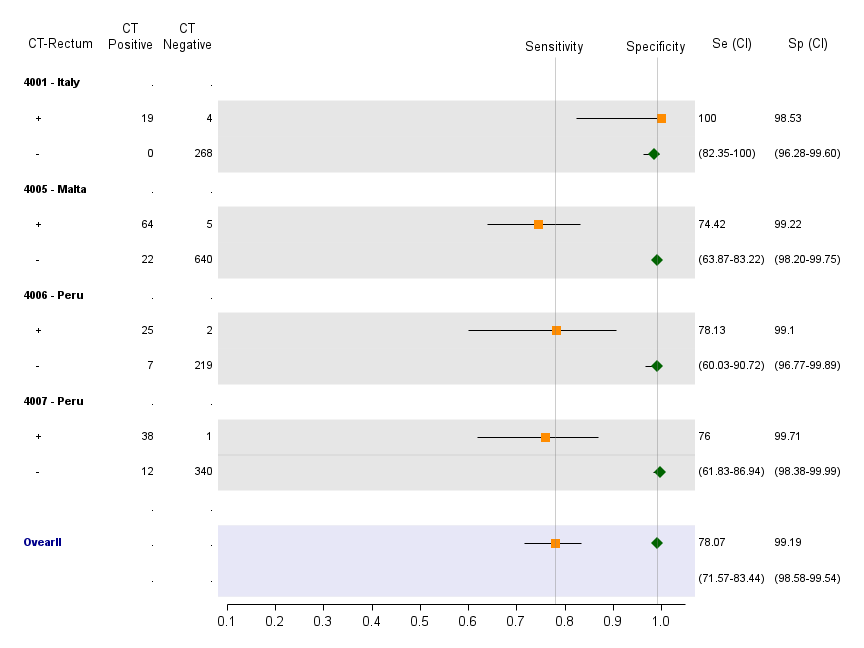 |
| **Urine** | 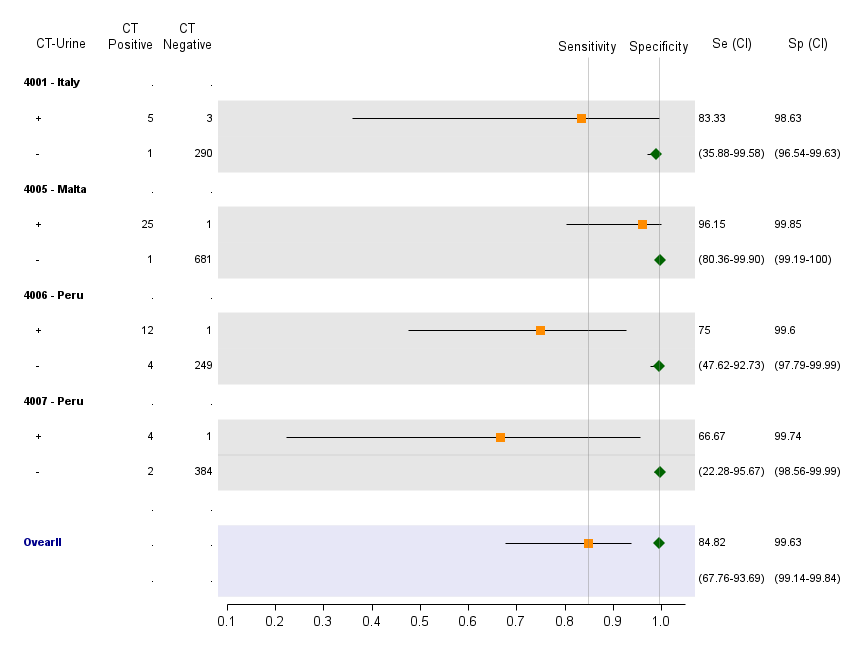 |

Table 4S: time study participants are willing to wait for POCT result, by geographical site**.**

|  |  | **Overall** | | **Italy** | | **Malta** | | **Peru (site 1)** | | **Peru (site 2)** | |
| --- | --- | --- | --- | --- | --- | --- | --- | --- | --- | --- | --- |
| **Variable** | **Category** | **N** | **%** | **N** | **%** | **N** | **%** | **N** | **%** | **N** | **%** |
| **How long would you be willing to wait?** | up to 20 min | 90 | 5.51 | 25 | 8.96 | 57 | 7.79 | 8 | 1.29 | 8 | 1.29 |
|  | up to 30 min | 413 | 25.29 | 50 | 17.92 | 220 | 30.05 | 143 | 22.99 | 143 | 22.99 |
|  | up to 1 hour | 679 | 41.58 | 102 | 36.56 | 319 | 43.58 | 258 | 41.48 | 258 | 41.48 |
|  | up to 2 hours | 366 | 22.41 | 77 | 27.60 | 92 | 12.57 | 197 | 31.67 | 197 | 31.67 |
|  | Don't know | 44 | 2.69 | 7 | 2.51 | 33 | 4.51 | 4 | 0.64 | 4 | 0.64 |
|  | Other | 41 | 2.51 | 18 | 6.45 | 11 | 1.50 | 12 | 1.93 | 12 | 1.93 |
|  | Missing | 69 |  | 20 |  | 10 |  | 39 |  | 39 |  |

*Note: missing values are not included in the denominator*
